# Circadian gating of dark‐induced increases in chloroplast‐ and cytosolic‐free calcium in Arabidopsis

**DOI:** 10.1111/nph.16280

**Published:** 2019-11-22

**Authors:** María Carmen Martí Ruiz, Hyun Ju Jung, Alex A. R. Webb

**Affiliations:** ^1^ Department of Stress Biology and Plant Pathology CEBAS‐CSIC Campus Universitario de Espinardo Murcia 30100 Spain; ^2^ Department of Plant Sciences University of Cambridge Downing Street Cambridge, CB2 3EA UK

**Keywords:** Arabidopsis, calcium signalling, chloroplast, circadian clock, cytosol, light–dark transition

## Abstract

- Changes in the spatiotemporal concentration of free Ca^2+^ ([Ca^2+^]) in different organelles of the cell contribute to responses of plants to physiological and environmental stimuli. One example are [Ca^2+^] increases in the stroma of chloroplasts during light‐to‐dark transitions; however, the function and mechanisms responsible are unknown, in part because there is a disagreement in the literature concerning whether corresponding dark‐induced changes in cytosolic [Ca^2+^] ([Ca^2+^]_cyt_) can be detected.
- We have measured changes in [Ca^2+^]_cyt_ upon darkness in addition to the already known dark‐induced increases in [Ca^2+^]_stroma_ in the aerial part of the *Arabidopsis thaliana* plant.
- These [Ca^2+^]_cyt_ transients depend on the photoperiod and time of day, peaking at anticipated dusk, and are superimposed on daily 24 h oscillations in [Ca^2+^]_cyt_. We also find that the magnitude of the dark‐induced increases in Ca^2+^ in both the cytosol and chloroplasts are gated by the nuclear circadian oscillator.
- The modulation of the magnitude of dark‐induced increases in [Ca^2+^]_stroma_ and [Ca^2+^]_cyt_ by transcriptional regulators in the nucleus that are part of the circadian oscillator demonstrates a new role for the circadian system in subcellular Ca^2+^ signalling, in addition to its role in driving circadian oscillations of [Ca^2+^] in the cytosol and chloroplasts.

## Introduction

A wide range of plant cell responses to environmental stimuli are associated with specific changes in the spatiotemporal concentration of free Ca^2+^ ([Ca^2+^]), known as ‘Ca^2+^ signatures’, present in numerous cellular types and compartments (McAinsh *et al.*, 1995; Kiegle *et al.*, 2000; Martí *et al.*, 2013; Sello *et al.*, 2018). In the cytosol, Ca^2+^ signatures arise from fluxes of Ca^2+^ into the cytosol across the plasma membrane or by release from internal stores. Downstream pathways decode the Ca^2+^ signatures to allow the cell to respond appropriately (Dodd *et al.*, 2010).

The timescale over which the Ca^2+^ signatures can occur varies greatly. Abiotic and biotic signals can cause rapid increases in [Ca^2+^]_cyt_ (Lynch *et al.*, 1989; Price *et al.*, 1994; Knight *et al.*, 1997; Monshausen *et al.*, 2009). On a slower scale, there are 24 h [Ca^2+^]_cyt_ daily rhythms, regulated by the circadian clock and light signalling (Dalchau *et al.*, 2010). Diel oscillations of [Ca^2+^]_cyt_ in light and dark cycles, or constant light, rise to a peak of *c.* 300 nM towards the middle and end of the photoperiod (Johnson *et al.*, 1995; Love *et al.*, 2004; Dalchau *et al.*, 2010). Circadian oscillations of [Ca^2+^]_cyt_ occur predominantly in the spongy mesophyll cells (Martí *et al.*, 2013), are driven by the rhythmic production of cyclic ADP ribose (Dodd *et al.*, 2007) and are suppressed specifically by the circadian oscillator gene *CIRCADIAN CLOCK ASSOCIATED 1* (*CCA1*) (Dodd *et al.*, 2007). Daily and circadian oscillations of [Ca^2+^]_cyt_ form part of the circadian oscillator, regulating its function through sensing by CALMODULIN‐LIKE 24, a Ca^2+^ sensor protein that interacts genetically with the circadian oscillator protein TIMING OF CAB 1 (TOC1) (Martí *et al.*, 2018).

Similar to the cytosol, there are chloroplastic circadian [Ca^2+^] oscillations, in addition to increases in chloroplast stromal [Ca^2+^] ([Ca^2+^]_stroma_) in response to biotic and abiotic signals (Johnson *et al.*, 1995; Nomura *et al.*, 2012; Sello *et al.*, 2016). Changes in [Ca^2+^] in the chloroplasts regulate aspects of photosynthesis, organelle division and the import of nuclear‐encoded proteins (Rocha and Vothknecht, 2012; Nomura and Shiina, 2014; Hochmal *et al.*, 2015). Additionally, the environmental transition between light and darkness produces a prolonged and sustained increase in [Ca^2+^]_stroma_ (Sai & Johnson, 2002; Nomura *et al.*, 2012; Sello *et al.*, 2016; Loro *et al.*, 2016) that depends on photoperiod. However, it was concluded that these dark‐induced increases in [Ca^2+^]_stroma_ are not modulated (or ‘gated’) by the nuclear circadian oscillator (Sai & Johnson, 2002).

Many studies have tried to unravel the mechanisms and biological role that underlie the generation and dissipation of [Ca^2+^] transients in the chloroplasts upon darkness (Sai & Johnson, 2002; Loro *et al.*, 2016; Sello *et al.*, 2018; Frank *et al.*, 2019). Recently, new Arabidopsis lines expressing Aequorin in different chloroplastic compartments have been developed (Sello *et al.*, 2018) and two Arabidopsis chloroplast‐targeted Ca^2+^ transporters, BIVALENT CATION TRANSPORTER 1 (BICAT1) and BICAT2, have been found to determine the amplitude of the dark‐induced [Ca^2+^]_stroma_ increase (Frank *et al.*, 2019). The later study suggested that the most straightforward explanation for the strong diminution of the *bicat2* mutant [Ca^2+^]_stroma_ transients is a dark‐triggered influx of Ca^2+^ from the cytosol. However, the authors reported that this idea is currently not favoured because [Ca^2+^]_cyt_ recordings failed to detect a consistent decrease of [Ca^2+^]_cyt_ upon the onset of darkness (Sai & Johnson, 2002; Nomura *et al.*, 2012; Sello *et al.*, 2016, 2018); this has therefore led to the hypothesis that the generation of the dark‐induced [Ca^2+^]_stroma_ signal is a result of Ca^2+^ being released from a hypothetical chloroplastic store. This hypothesis is also supported because buffering cytosolic Ca^2+^ with the chelator EGTA combined with digitonin had no effect on Ca^2+^ transients in the chloroplasts (Loro *et al.*, 2016). However, short stromal [Ca^2+^] spikes were strongly reduced, suggesting that cytosolic Ca^2+^ and/or cellular integrity are the source of, or at least necessary for, the spikes (Loro *et al.*, 2016). Therefore, more studies are necessary to understand how and why the chloroplastic [Ca^2+^] transients upon darkness are generated.

The nuclear circadian oscillator can regulate photosynthetic activity in the chloroplast, at least in part because, in Arabidopsis, the nuclear‐encoded SIGMA FACTOR5 (SIG5) controls circadian rhythms of transcription of several chloroplast genes (Noordally *et al.*, 2013). Because the nuclear circadian oscillator can regulate events in the plastids, we decided to revisit whether the generation of the dark‐induced [Ca^2+^]_stroma_ signal was gated by the circadian clock, in order to understand how this signal is controlled. In parallel, we have investigated the regulation of Ca^2+^ signals in the cytosol by light‐to‐dark transitions to resolve a debate in the literature about the potential mechanisms for the regulation of plastid Ca^2+^ transients.

Here, we demonstrate that similar to the chloroplasts, there are reproducible and consistent increases in [Ca^2+^]_cyt_ at the onset of darkness in the aerial part of the plant, which are superimposed on the daily 24 h oscillations in [Ca^2+^]_cyt_. We report that dark‐induced transient in [Ca^2+^] in both the cytosol and stroma are gated by the circadian clock. This finding demonstrates an important new role for the circadian system in subcellular Ca^2+^ signalling, and also establishes a new link between eukaryotic circadian clocks and organelles of endosymbiotic origin.

## Materials and Methods

### Plant materials and growth conditions

*Arabidopsis thaliana* ecotypes Columbia‐0 (Col‐0), Wassilewskija‐2 (Ws‐2), Landsberg erecta (L*er*) and *cry1*, *cry2*, *phyA* and *phyB* mutants carrying *CaMV 35S:AEQUORIN (35S:AEQ)* were described previously in Xu *et al.* (2007) and Sai & Johnson (2002). *prr7*‐11 *prr5*‐10 *prr9*‐11 (Nakamichi *et al.*, 2005) plants carrying *35S:AEQ* targeted to the cytosol were obtained as described in Xu *et al.* (2007).

Arabidopsis seeds were surface‐sterilized with 10% (v/v) NaClO and 0.1% (v/v) Triton X‐100 for 3 min and rinsed three times with sterile dH_2_O. Surface‐sterilized seeds were sown onto 0.8% (w/v) bactoagar plates containing ½ Murashige & Skoog (½MS) medium (pH 5.7 with 0.5 M KOH) without sucrose and stratified in the dark for 48 h at 4°C. Seeds were germinated and entrained in growth cabinets (Sanyo, Bracknell, UK) with a constant temperature of 19°C and 100 μmol m^−2^ s^−1^ cool white light from fluorescent tubes under 12 h : 12 h, light : dark cycles, unless otherwise stated.

### Aequorin imaging for dark‐induced [Ca^2+^] transient using ICCD225 photon‐counting camera system

Photon counting was performed in a light‐tight box using an ICCD225 photon‐counting camera system (Photek, Hastings, UK) mounted above the seedlings. The camera chambers supplied equal amounts of red (630 nm)/blue (470 nm) LED light in a mixed array (100 μmol m^−2^ s^−1^) at the desired photoperiod and was cooled to 19–20°C. When just red or blue light was used, the intensity was 50 μmol m^−2^ s^−1^. Luminescence was recorded from clusters of seven to 12 seedlings grown as described, and the data for one experiment were obtained as the sum of the signal of all the cluster together. Image analysis was done with Photek ifS32 software.

For measurements lasting > 1 d or for one‐time point measurements, photon‐counting images were captured every 2 h for 1500 s following a wait of 200 s post‐illumination to allow light from delayed fluorescence to scatter or at the end of the photoperiod for 7000 s or a different time point when stated, respectively. In both, seedlings were incubated with 50 μl of 20 µM coelenterazine (Prolume, Pinetop, AR, USA) for 20 min in the dark the night before going into the camera box when they were 11–12 d old.

### Estimation of daily and circadian oscillations of [Ca^2+^]_cyt_

Estimation of daily and circadian oscillations of [Ca^2+^]_cyt_ was performed as described by Love *et al.* (2004). Sixteen‐bit images of the photon density generated from the 1500 s or the last 700 s of each integration were obtained and processed using Photek ifs32. Circadian parameters were analysed using the brass plug‐in for ms excel (http://www.amillar.org) to carry out fast Fourier transform nonlinear least‐squares analysis (Plautz *et al.*, 1997) with period limits between 18 and 35 h at 95% confidence level. Rhythms were analysed for at least three cycles in constant conditions after the first 24 h. Traces with a relative amplitude error > 0.5 were considered arrhythmic.

### Aequorin imaging for dark‐induced [Ca^2+^] transient using a luminometer

Measurement of bioluminescence from the Ca^2+^ reporter aequorin and calibration to estimate [Ca^2+^] were done as follows. Arabidopsis seedlings were grown as described for the camera system. When plants were 11–13 d old, three plants were placed within a luminometer tube (51 mm long × 12 mm diameter; Sarstedt, Leicester, UK) containing 1 ml of 0.8% (w/v) bactoagar media with ½MS and incubated with 100 μl of 20 µM coelenterazine (Prolume) for 20 min in the dark. At the end of the photoperiod, bioluminescence was measured using a photon‐counting luminometer (photomultiplier tube 9899A) cooled to −20ºC with a FACT50 housing (Electron Tubes, Uxbridge, UK) (Martí *et al.*, 2013). Aequorin bioluminescence was captured every second for at least 2 h and finally discharged by 1 ml of discharge solution (2 M CaCl_2_ dissolved in 20% (v/v) ethanol). Measurements were made until the detected luminescence reached 10% of the first peak after discharge injection. [Ca^2+^] concentrations were determined according to Fricker *et al.* (1999).

### Statistical analysis

*F*‐test two‐sample for variances followed by two‐tailed Student’s *t*‐test were performed to compare the changes in area under the aequorin luminescence curves from plants transferred from light to darkness or plants that were in the dark for 6 h.

## Results

### Changes in cytosolic free calcium in Arabidopsis upon darkness

**Figure 1:**
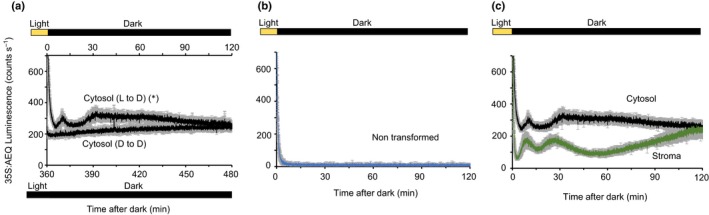
Dark‐induced [Ca^2+^]_cyt_ transient occurs after light off in 12 h : 12 h, light : dark cycles. Luminescence from reconstituted aequorin in Arabidopsis seedlings expressing aequorin targeted to the cytosol (black) (a, c) or the stroma (green) (c) and from nontransgenic seedlings (blue) (b). The ecotypes used were Columbia‐0 (Col‐0) and Wassilewskija‐2 (green) (c). Data were recorded immediately after plants were transferred to darkness at the end of the photoperiod (12 h after the lights went on) or after being 6 h in the dark (a; D to D) as shown in the bars above or below the figures. Data are the mean luminescence ± SE of eight (a; L to D), four (a; D to D), two (b) and three (c; stroma) experiments. Each experiment consisted of 24 clusters of seedlings, and each cluster contained 7 to 10 seedlings. See Supporting Information Figs 1, 2, 3 for the replicates. In (a): *, *P* < 0.05 (Student’s *t*‐test for equal variances of the areas under the traces).

Using an ICCD225 photon‐counting camera to detect luminescence of aequorin in the cytoplasm, we detected a very prolonged increase in luminescence at 3.5 min after the plants were transferred from white light to darkness at the end of the photoperiod (12 h after the onset of light) (Fig. 1a; Supporting Information Fig. S1). The dark‐induced [Ca^2+^]_cyt_ transient at dusk could be divided in two phases. First, we observed a ‘spike’ that peaks at *c.* 10 min after the onset of darkness and, second, a sustained increase that peaks at *c.* 30 min after the start of darkness and decays over the next 2 h. During the first 3.5 min, aequorin luminescence is contaminated by photons originating from delayed Chl fluorescence and as a consequence, aequorin luminescence and therefore changes in [Ca^2+^]_cyt_ immediately following the light‐to‐dark transition could not be detected. Plants that were recorded 6 h into the dark period did not have any change in luminescence (Figs 1a, S2). Statistical analysis of the area under the curve for the luminescence traces in plants that were transferred from light to darkness and plants that were already in the dark for 6 h revealed significant differences between the two conditions studied (light to dark, 10 453 ± 1359 photon counts; dark for 6h, 7321 ± 203 photon counts; Student’s *t*‐test for equal variances, *P* = 0.04, df = 8, *t* = 2.26), suggesting that it is the transfer from light to dark that results in the change of aequorin luminescence. No signal was detected from nontransgenic Col‐0 plants (Figs 1b, S2) grown and assayed using the same method as the transgenic plants (Fig. 1a), including treatment with coelenterazine, allowing us to conclude that the increase in luminescence that occurs from 3.5 min after darkness is a result of changes in aequorin luminescence and therefore [Ca^2+^]_cyt_.

The dark‐induced [Ca^2+^]_cyt_ signal measured using the cytosolic aequorin in plants that were grown in 12 h : 12 h, light : dark cycles differs from the dark‐induced signal from aequorin targeted to the stroma (Sai & Johnson, 2002). Comparison of the cytosolic and stromal [Ca^2+^] signals in Fig. 1(c) shows that the prolonged [Ca^2+^]_cyt_ signature does not mimic the [Ca^2+^]_stroma_ signature, mostly in the sustained increase of the cytosolic signal that peaks around 30 min after the start of darkness and decays over the next 2 h, whereas the stromal [Ca^2+^] signal consists of two peaks at *c. *8 and 25 min, lasting 40 min, followed by a prolonged increase that lasts about 2 h after dark (Figs 1c, S3). The difference in dynamics between the luminescence reported by the stromal‐ and cytosolic‐targeted aequorin suggests that the two signals are distinct, emanating from different compartments, and that there are specific dark‐induced increases in [Ca^2+^]_cyt_. Interestingly, the dark‐induced [Ca^2+^]_stroma_ spike measured by the photon‐counting camera was slightly different from those measured previously by others (Sai and Johnson 2002; Nomura *et al.*, 2012; Sello *et al.*, 2016, 2018) and ourselves (Fig. S3) in a luminometer, consisting of a rapid increase in [Ca^2+^]_stroma_ that reaches a peak between 25 min after dusk and decays close to basal values within the next 2 h. Thus, we conclude that there is an increase in [Ca^2+^]_cyt_ in response to darkness, which was not detected in some of the previous studies (Nomura *et al.*, 2012; Sello *et al.*, 2016, 2018).

We were unable to calibrate the magnitude of the increase in [Ca^2+^]_cyt_ measured using intensified charge‐coupled device (CCD) arrays because the discharge of available aequorin with excess Ca^2+^ to perform a normalization saturated pixels on the CCD detector. To obtain an estimate of the magnitude of the dark‐induced increase in [Ca^2+^]_cyt_ that occurs at 3.5 min after darkness, when Chl fluorescence has dissipated, we revisited the use of luminometry in the same experimental conditions, with three plants per sample to avoid the leaves covering each other (Fig. S4). In 12 out of 14 experiments we detected changes in [Ca^2+^]_cyt_ after the transition into darkness. These changes peaked at *c. *10 min after darkness and lasted 10 min with a variable estimated amplitude of 5–65 nM [Ca^2+^]_cyt_. This might be an underestimate because the normalization assumes all cells contribute equally to the response. The signature detected with the luminometer in the cytosol (Fig. S4) was broadly like the one detected in the camera but contained less detail (Figs 1a, S1). For the cytosolic signal, the sustained increase peaking at *c. *30 min and decaying over the next 2 h in darkness was not detected using luminometry.

### The dark‐induced cytosolic‐free calcium transient emanates from green tissue

**Figure 2:**
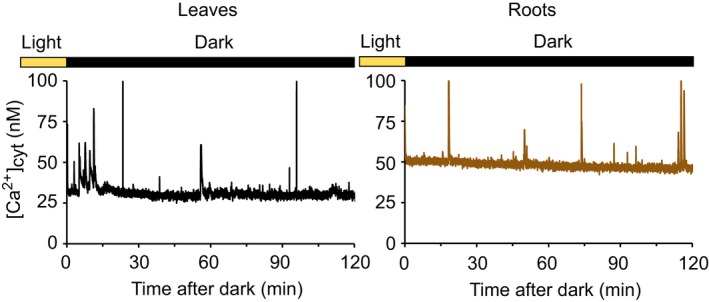
The dark‐induced [Ca^2+^]_cyt_ transient occurs in green tissues. Three Col‐0 Arabidopsis transgenic seedlings expressing aequorin targeted to the cytosol were grown in white light : dark cycles. On the night of the 11^th^ day of growth, the root and leaves were separated and incubated with coelenterazine. Aequorin luminescence was recorded in a luminometer from reconstituted aequorin when the tissues were 12 d old. Traces represent the data obtained in one experiment. Experiments were repeated four times.

To determine from where in the plant the dark‐induced [Ca^2+^]_cyt_ transient arises, we performed a luminometry experiment using excised green tissue or roots, to separate the signal coming from the two tissues during the data acquisition. When the luminescence from the two different tissues was analysed separately, the dark‐induced [Ca^2+^]_cyt_ increase was detected in the green tissue and no increase was observed in the roots (Figs 2, S5).

### Identification of the signalling pathways leading to dark‐induced cytosolic‐free calcium increases

**Figure 3:**
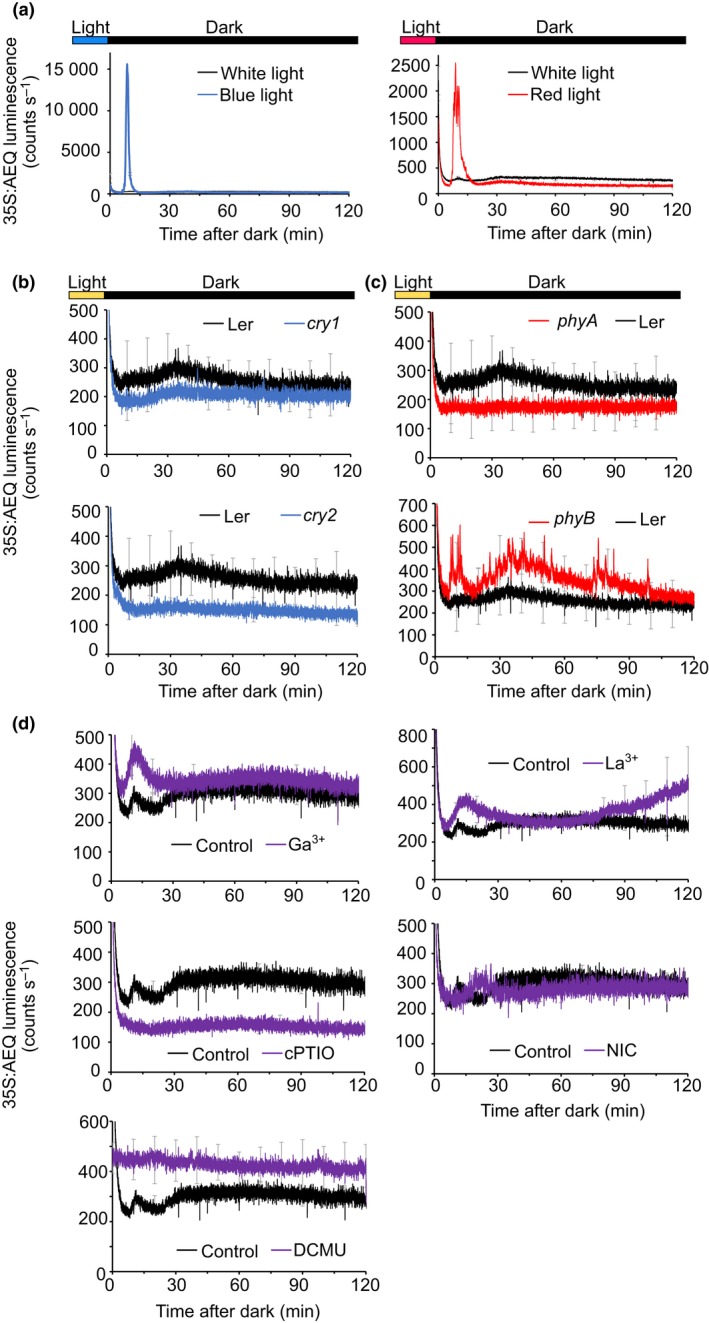
Identification of the signalling pathways leading to the cytosolic‐free calcium transient induced by dark. Luminescence from reconstituted aequorin in Landsberg erecta (L*er*), *cry1*, *cry2*, *phyA* and *phyB* mutants and Col‐0 Arabidopsis seedlings expressing aequorin targeted to the cytosol was recorded immediately after plants were transferred to darkness at the end of the photoperiod (12 h after the lights went on), as shown in the bars above the figures. In (a) plants were grown in white light and transferred to monochromatic red or blue light 4 d before the measurements. In (b–d), all treatments were applied 30 min before sunset, but nicotinamide was applied when plants were 7 d old and 3‐(3,4‐dichlorophenyl)‐1,1‐dimethylurea (DCMU) was applied 24 h before measurements. Data are the mean luminescence ± SE of three experiments consisting of 24 clusters of seedlings, each cluster containing 7 to 10 seedlings. Error bars are indicated every 10 min for clarity.

As the dark‐induced increase in [Ca^2+^]_cyt_ was dependent on prior illumination (Fig. 1a) we tested whether a particular photoreceptor system is involved in the perception of light before darkness. Growth of plants in either monochromatic red or blue light alone allowed the [Ca^2+^]_cyt_ changes upon darkness, suggesting that the [Ca^2+^]_cyt_ change that occurs when plants are transferred from light to darkness involves both red and blue photoreceptor systems (Fig. 3a). Monochromatic light resulted in a larger spike at 10 min than seen when plants were grown in white light, although we are uncertain why this might be.

To test further the involvement of the red and blue photoreceptors, we performed a light‐to‐dark transition using *cry1*, *cry2*, *phyA* and *phyB* Arabidopsis mutants. Statistical analysis of the changes in the area under the curve for the luminescence comparing *cry* mutants with wild‐type (WT) plants (Fig. 3b) resulted in no significant differences (L*er*, 8135 ± 906; *cry1* 6677 ± 60 (Student’s *t*‐test for unequal variances, *P* = 0.32, df = 2, *t* = 4.3); L*er*, 8135 ± 906; *cry2*, 4676 ± 132 (*P* = 0.09, df = 2, *t* = 4.3)). These analyses indicated that the dark‐induced [Ca^2+^]_cyt_ changes were still present in these mutant lines, suggesting that the remaining photoreceptor systems in the mutants were able to respond to the transition into darkness. In Fig. 3(c) we tested the effect of the red light photoreceptor mutants *phyA* and *phyB* on the dark‐induced [Ca^2+^]_cyt_ changes. The absence of *PHYB* did not affect the response (L*er*, 8135 ± 906; *phyB*, 14 117 ± 1639 (Student’s *t*‐test for equal variances, *P* = 0.06, df = 4, *t* = 2.77)). However, we found that in the *phyA* mutant, the dark‐induced [Ca^2+^]_cyt_ was absent (L*er* 8135 ± 906, *phyA* 3837 ± 332 (*P* = 0.02, df = 4, *t* = 2.77)), suggesting that *PHYA*, at least, is directly involved in the light signalling pathway that leads to the dark‐induced cytosolic‐free calcium increases.

Additionally, we performed a set of experiments to investigate whether the alteration of [Ca^2+^]_cyt_ after darkness was derived from internal stores or from the extracellular space. First, we decided to evaluate whether Ca^2+^ influx to the cytosol might be across the plasma membrane using two inhibitors of plasma membrane‐mediated influx of Ca^2+^, 1 mM GdCl_3_ and 10 mM LaCl_3_ (Véry & Davies, 2000), which were added 30 min before the measurement of [Ca^2+^]_cyt_ at the end of the photoperiod. The dark‐induced [Ca^2+^]_cyt_ transient was insensitive to Gd^3+^ and La^3+^ when the area changes under the traces were determined (control, 12 844 ± 642; Ga^3+^, 12 406 ± 1657 (Student’s *t*‐test for equal variances, *P* = 0.79, df = 3, *t* = 3.18); control, 12 844 ± 642, La^3+^, 16 816 ± 7296 (*P* = 0.68, df = 2, *t* = 12.71)) (Fig. 3d), suggesting that the primary pathway by which dark increases [Ca^2+^]_cyt_ in Arabidopsis might be from an intracellular compartment. Cyclic ADP ribose (cADPR) is a Ca^2+^‐signalling molecule synthesized by ADP‐ribosyl cyclase (ADPRc) which can release Ca^2+^ into the cytosol from the endoplasmic reticulum (ER) and vacuole (Leckie *et al.*, 1998; Navazio *et al.*, 2000; Sánchez *et al.*, 2004). ADPRc activity and cADPR can be detected, and the enzymatic protein has been recently identified (Dodd *et al.*, 2007; Abdul‐Awal *et al.*, 2016; Wan *et al.*, 2019). cADPR is thought to regulate circadian oscillations of [Ca^2+^]_cyt_ (Dodd *et al.*, 2007). We tested for the potential involvement of cADPR using nicotinamide, an ADPRc activity inhibitor, and cPTIO, a nitric oxide (NO) scavenger, because NO increases ADPRc activity (Abdul‐Awal *et al.*, 2016). When plants were incubated with 20 mM nicotinamide from the age of 7 d, or 0.3 mM cPTIO, which was added 30 min before measurement, the dark‐induced [Ca^2+^]_cyt_ transient was abolished or decreased (control, 12 844 ± 642; nicotinamide, 9583 ± 451 (Student’s *t*‐test for equal variances, *P* = 0.04, df = 3, *t* = 3.18); control, 12 844 ± 642; cPTIO, 5953 ± 49 (*P* = 0.009, df = 2, *t* = 4.30)) (Fig. 3d), suggesting that ADPRc activity might be necessary for the dark‐induced [Ca^2+^]_cyt_ signal. Additionally, we added 20 µM DCMU, an inhibitor of the photosynthetic electron transport chain, 24 h before measurement, to test whether photosynthesis was involved in the generation of the [Ca^2+^]_cyt_ signal upon darkness. The addition of DCMU negatively affected the dark‐induced [Ca^2+^]_cyt_ increase (control, 12 844 ± 642; DCMU, 8991 ± 885; Student’s *t*‐test for equal variances, *P* = 0.02, df = 4, *t* = 2.77), suggesting that photosynthesis may affect the [Ca^2+^]_cyt_ signal upon darkness (Fig. 3d).

### Dark‐induced transients in cytosolic‐free calcium might encode information about photoperiod and time of day

**Figure 4:**
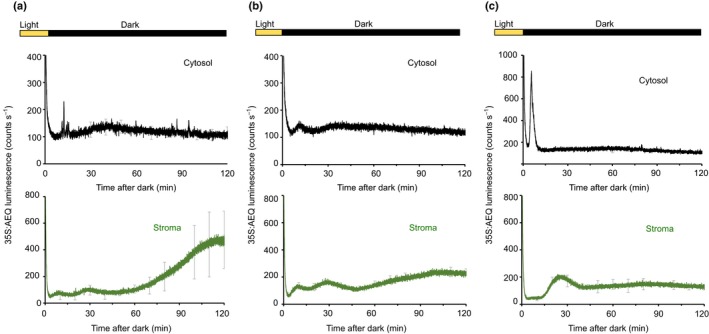
Dark‐induced [Ca^2+^]_cyt_ signature is affected by photoperiod length. Luminescence from reconstituted aequorin in Col‐0 (black) or Wassilewskija‐2 (Ws‐2, green) Arabidopsis seedlings expressing aequorin targeted to the cytosol (black) or the stroma (green). Plants were grown in white light : dark cycles (a, 8 h : 16 h; b, 12 h : 12 h; c, 16 h : 8 h) (100 µmol m^−2^ s^−1^) for 12–13 d. Data were recorded immediately after plants were transferred to darkness at the end of the photoperiod (8 h (a), 12 h (b) and 16 h (c) after the lights went on). Data are the mean luminescence ± SE of three experiments consisting of 24 clusters of seedlings, each cluster containing 7 to 10 seedlings. Error bars are indicated every 10 min for clarity.

Transition to darkness is the laboratory mimic of end of day at the onset of night. Therefore, we tested the hypothesis that changes in [Ca^2+^]_cyt_ upon darkness might encode information about day length. We grew plants under different photoperiods, such as long‐day cycles (LD, 16 h : 8 h, light : dark) and short‐day cycles (SD, 8 h : 16 h, light : dark). The [Ca^2+^] response in the cytosol was affected by the length of day (Fig. 4). The dark‐induced [Ca^2+^]_cyt_ changes were very similar in plants grown in SD and 12 h : 12 h, light : dark cycles (Fig. 4a,b). However, when plants were grown in LD conditions, the [Ca^2+^]_cyt_ signature was different, showing a larger peak at 7 min which lasted for 5 min before returning to the basal value (Fig. 4c). We also examined whether the dark‐induced [Ca^2+^]_stroma_ signal was sensitive to day length. It has been reported that in LD conditions, the [Ca^2+^]_stroma_ peaks lasted for 1 h and then returned to the basal value, whereas in SD conditions, the profile of Ca^2+^ [Ca^2+^]_stroma_ included a smaller peak later in the night that was not observed in the LD photoperiod (Sai & Johnson, 2002). We observed similar results under LD conditions (Fig. 4c), where a [Ca^2+^]_stroma_ increase was present at *c.* 25 min after darkness. However, in SD conditions we observed a later, large and prolonged peak that is not present in plants grown under LD conditions (Fig. 4a). Finally, when we compared the differences and similarities in the dynamics between the cytosolic and stromal signals, we found that the differences between the two signals are more apparent in SD and LD conditions than in a 12 h : 12 h, light : dark cycle (Fig. 4). The different responses of the cytosolic and stromal signals to the change in photoperiod might suggest a different mechanistic basis.

**Figure 5:**
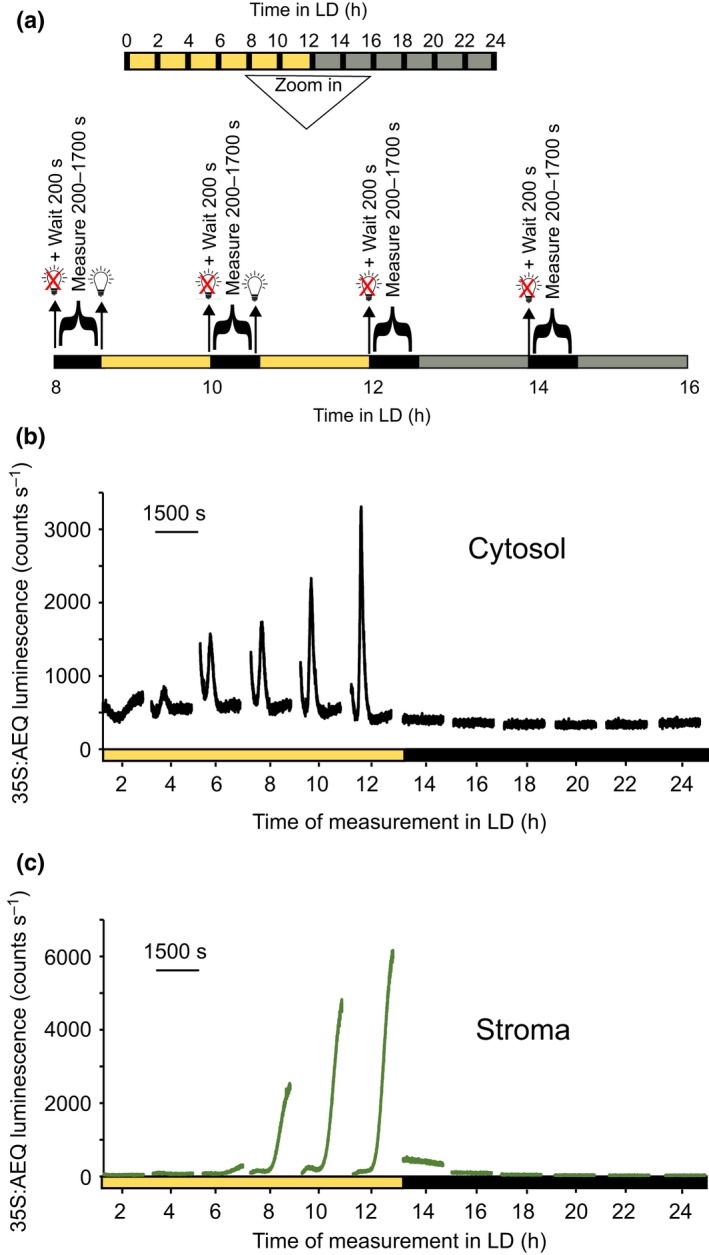
The dark‐induced [Ca^2+^]_cyt_ transient depends on the time of the day. Arabidopsis transgenic seedlings expressing aequorin targeted to the cytosol (Col‐0) or the stroma (Wassilewskija‐2, Ws‐2) were grown in white light : dark cycles (12 h : 12 h) (100 µmol m^−2^ s^−1^). On the night of the 11^th^ day of growth, seedlings were incubated with coelenterazine, and aequorin luminescence was recorded from reconstituted aequorin from 12 d of age, as shown in (a). (b, c) Data of changes in [Ca^2+^]_cyt_ and [Ca^2+^]_stroma_, respectively, every 2 h during one light and dark cycle. The data represent one experiment consisting of 80 clusters of seedlings, each cluster containing 7 to 10 seedlings. Experiments were repeated at least six times (b) or twice (c).

**Figure 6:**
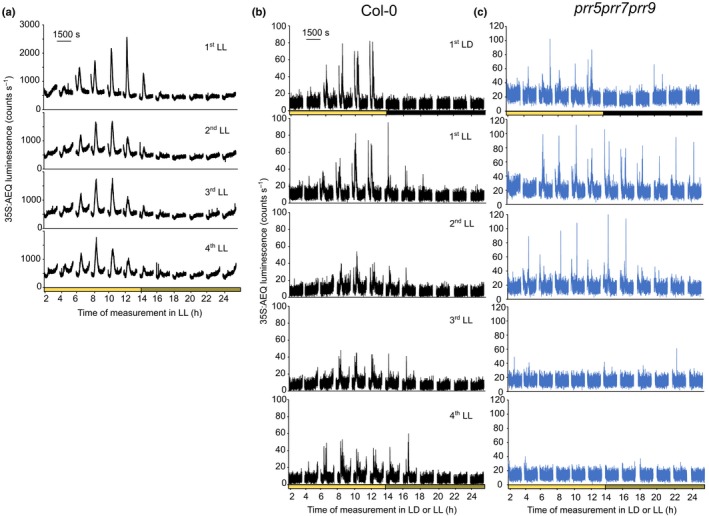
The dark‐induced [Ca^2+^]_cyt_ transient is gated by the circadian clock. Col‐0 and *prr7*‐11 *prr5*‐10 *prr9*‐11 triple mutant Arabidopsis transgenic seedlings expressing aequorin targeted to the cytosol were grown in white light : dark cycles (12 h : 12 h) (100 µmol m^−2^ s^−1^). On the night of the 11^th^ day of growth, seedlings were incubated with coelenterazine, and aequorin luminescence was recorded from reconstituted aequorin from 12 d of age. (a) Data of changes in [Ca^2+^]_cyt_ every 2 h during constant light (LL) cycles in Col‐0 plants. (b, c) Data of changes in [Ca^2+^]_cyt_ every 2 h during one light and dark, and four LL cycles in Col‐0 and *prr7*‐11 *prr5*‐10 *prr9*‐11 triple mutant plants, respectively. Data represent one experiment consisting of 80 (a) or eight (b, c) clusters of seedlings, each cluster containing 7 to 10 seedlings. Experiments were repeated at least seven times (a) or twice (b).

As there was an effect of length of photoperiod on the dark‐induced [Ca^2+^]_cyt_ signal, we tested whether the magnitude of the Ca^2+^ increase was dependent on the time of transfer to darkness. We compared the photon‐counting data captured at different times of the day from plants that were growing in mixed red and blue light‐dark cycles (12 h : 12 h, light : dark). Data were recorded every 2 h over 1 d in 12 h: 12 h conditions as shown in Fig. 5(a). If plants were in the photoperiod phase, every 2 h they were transferred to darkness and data were recorded for 1500 s following a wait of 200 s post‐illumination to allow light from delayed fluorescence to scatter. After each measurement, lights were turned on and remained on until the next measurement. When plants were in the dark period of the 24 h 12 h : 12 h, light : dark cycles, data were recorded every 2 h for 1500 s. The changes in [Ca^2+^]_cyt_ upon darkness were modulated by the time of day, being higher at the end of the photoperiod and absent during the night when there was no transition from light to darkness (Figs 5b, 6a,b). Similar behaviour was observed for the [Ca^2+^]_stroma_ (Figs 5c, S6). The absence of signal in nontransgenic plants treated with coelenterazine demonstrated that the time‐of‐day change in luminescence was a result of changes in [Ca^2+^] (Fig. S7).

The pattern of the [Ca^2+^]_cyt_ signature was different if plants were transferred to darkness every 2 h (Fig. 5b) or only at the end of the photoperiod (Fig. 1a). At dusk, the sustained increase observed in the camera after the first transient spike (Fig. 1a) was absent if plants were assayed every 2 h (Fig. 5b). The difference between these signals suggests there is some effect of integrated amount of light on the pattern of the signal, in addition to that of time of day.

### The dark‐induced cytosolic‐ and stroma‐free calcium transients are modulated by the circadian clock

The time of day‐dependent gating of the dark‐induced [Ca^2+^]_cyt_ increase suggests that the circadian timekeeper could be involved. To test this hypothesis, we measured dark‐induced Ca^2+^ increases in plants maintained without the prolonged darkness of night (i.e. constant light (LL) with dark interruptions, a protocol standard for circadian luminescence such as promoter::luciferase fusions measurements, because the short dark breaks do not interfere with functioning of the circadian oscillator (Millar *et al.*, 1995)). Similar to LD cycles, from the second day in LL during the subjective mornings, the dark‐induced [Ca^2+^]_cyt_ increase was detected, being higher in the later phases of the circadian subjective days, demonstrating that [Ca^2+^]_cyt_ can respond to darkness in the absence of the dark of night (Col‐0; Fig. 6a,b). Additionally, this time of day‐dependent modulation of the magnitude of the dark‐induced increases in [Ca^2+^]_cyt_ in LL conditions demonstrates that the signal observed is a result of gating by the circadian oscillator rather than as a consequence of a compounding effect of the signal owing to multiple stimulation or initiated in the light itself. Thus, at 2 h after the onset of dark (14 h) in the LD cycle, there was no increase in [Ca^2+^]_cyt_ (Fig. 5b), whereas at 14 h in the first LL cycle, a dark‐induced increase of [Ca^2+^]_cyt_ was measured which was lower than the one detected at 12 h in the same condition (Col‐0; Fig. 6a,b), demonstrating that the changes in [Ca^2+^]_cyt_ are specific for the transition from light to darkness. To further investigate the circadian regulation of [Ca^2+^]_cyt_ upon darkness, we measured [Ca^2+^]_cyt_ in the circadian arrhythmic triple mutant *prr7*‐11 *prr5*‐11 *prr9*‐10 (Nakamichi *et al.*, 2005). In the LD cycle and the first day in LL there are no differences between the mutant and the WT in terms of gating; however, in free‐running conditions in which the mutant is arrhythmic, the mutant failed to gate the dark‐induced [Ca^2+^]_cyt_ response (Figs 6b, S8). These data demonstrate that modulation of dark‐induced increases of [Ca^2+^]_cyt_ is an output of the nuclear circadian oscillator.

**Figure 7:**
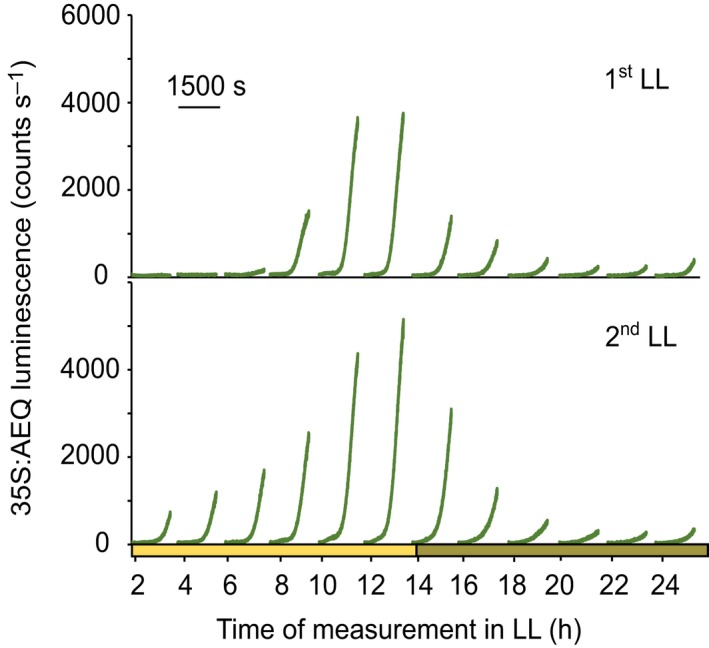
The dark‐induced [Ca^2+^]_stroma_ transient is gated by the circadian clock. Ws‐2 Arabidopsis transgenic seedlings expressing aequorin targeted to the stroma were grown in white light : dark cycles (12 h : 12 h) (100 µmol m^−2^ s^−1^). On the night of the 11^th^ day of growth, seedlings were incubated with coelenterazine, and aequorin luminescence was recorded from reconstituted aequorin from 12 d of age. On the 13^th^ day, plants were transferred to constant light (LL). The data show changes in [Ca^2+^]_stroma_ every 2 h during two LL cycles. The data represent one experiment consisting of 80 clusters of seedlings, each cluster containing 7 to 10 seedlings. Experiments were repeated at least twice.

A previous report concluded that dark‐induced regulation of [Ca^2+^]_stroma_ is not regulated by the nuclear circadian oscillator (Sai & Johnson, 2002). In that study, plants were transferred to the dark at different times after 5 d in constant light (Sai and Johnson, 2002). Because the circadian regulation of [Ca^2+^]_cyt_ damps over time, we decided to examine again whether [Ca^2+^]_stroma_ is under circadian control. We found that circadian gating persisted for at least two cycles in LL, suggesting that the dark‐induced [Ca^2+^]_stroma_ increase is modulated by the circadian oscillator in the nucleus and that the previous conclusion of no role for the circadian oscillator might be incorrect (Figs 7, S9).

### The dark‐induced transient of [Ca^2+^]_cyt_ is superimposed on daily and circadian [Ca^2+^]_cyt_ oscillations

Aequorin‐based luminescence measurements must be performed in the dark because the intensity of photons released by aequorin is many orders of magnitude lower than the light required in the standard growth conditions for plants. The method used here to determine the daily and circadian control of the dark‐induced [Ca^2+^]_cyt_ signal is the same one used to determine the daily and circadian [Ca^2+^]_cyt_ oscillations (Love *et al.*, 2004), which is based on standard protocols for measurement of circadian regulation of luminescent reporters such as promoter::luciferase fusions (Millar *et al.*, 1995). Taking this into account, as well as the finding that the circadian timekeeper gated the dark‐induced [Ca^2+^]_cyt_ increase, we decided to investigate whether the measured daily and circadian oscillations of basal [Ca^2+^]_cyt_ are a consequence of the measurement protocol or whether they occur in addition to the dark‐induced [Ca^2+^]_cyt_ increases.

**Figure 8:**
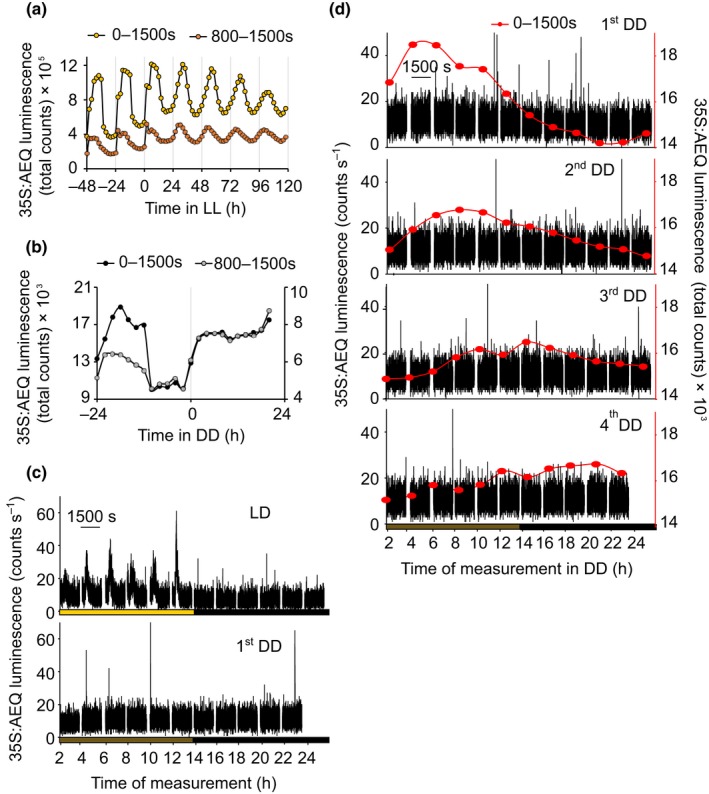
Dark‐induced transients in [Ca^2+^]_cyt_ are superimposed on daily and circadian [Ca^2+^]_cyt_ oscillations. (a) Daily and circadian [Ca^2+^]_cyt_ oscillations measured in constant light (LL) using different time integration intervals. Col‐0 Arabidopsis transgenic seedlings expressing aequorin targeted to the cytosol were grown in 12 h : 12 h, light : dark (LD) cycles and then transferred to LL. The data represent one experiment consisting of 80 clusters of seedlings and each cluster contained 7 to 10 seedlings. (b) Daily and circadian [Ca^2+^]_cyt_ oscillations measured in constant darkness (DD) without 3% (w/v) sucrose using different time integration intervals. (c) Changes in [Ca^2+^]_cyt_ recorded every 2 h and used in (b). (d) Daily and circadian [Ca^2+^]_cyt_ oscillations measured in DD with 3% (w/v) sucrose. The raw time series data are in black, showing no dark‐to‐light transitions. The integrated photon counts obtained during the 1500 s of recording are in red. (b–d) Col‐0 Arabidopsis transgenic seedlings expressing aequorin targeted to the cytosol were grown in 12 h : 12 h, light : dark cycles and then transferred to DD. (b–d) Data represent one experiment consisting of eight clusters of seedlings and each cluster contained 7 to 10 seedlings. Experiments were repeated at least six times (a) or (b–d) twice.

We used three different approaches to investigate the circadian control of basal [Ca^2+^]_cyt_. First, we measured the 24 h oscillations of [Ca^2+^]_cyt_ using the same method that we used to measure the daily and circadian control of the dark‐induced [Ca^2+^]_cyt_ signal (Figs 5, 6). We first attempted to separate the measurement of basal circadian [Ca^2+^]_cyt_ oscillations from the dark‐induced increase in [Ca^2+^]_cyt_ by investigating the signal generated by a dark break every 2 h in the photoperiod, because this protocol induces a dark Ca^2+^ signal that is not sustained. We compared the integrated photon counts obtained from 0 to 1500 s (including the dark‐induced [Ca^2+^]_cyt_ signal) and the last 800 s of each integration (not including the dark‐induced [Ca^2+^]_cyt_ signal) to determine if we could detect basal circadian [Ca^2+^]_cyt_ oscillations in addition to the dark‐induced signal. In both, the first 1500 s (representing dark‐induced and basal signals) and the last 800 s (representing only basal signals), [Ca^2+^]_cyt_ increased during the day, peaked several hours after dawn and then decreased, reaching the minimum value during the night (Figs 8a, S10). When the circadian parameters were determined, similar values were found for both signals (0–1500 s, period = 23.6 h, relative amplitude error (RAE) = 0.18; 800–1500 s, period = 23.6 h, RAE = 0.23).

We next investigated the circadian regulation of [Ca^2+^]_cyt_ in constant darkness (DD), a condition that does not have a light‐to‐dark transition during measurement but is complicated by the dampening of circadian rhythms of [Ca^2+^]_cyt_ in prolonged DD in Arabidopsis (Johnson *et al.*, 1995; Xu *et al.*, 2007). This dampening occurs after *c. *16 h in DD because the machinery that returns [Ca^2+^]_cyt_ to resting values does not operate in extended darkness (Xu *et al.*, 2007; Fig. 8b). The persistence of rhythmic [Ca^2+^]_cyt_ in the subjective morning of the first DD cycle suggests the presence of basal [Ca^2+^]_cyt_ oscillations that are not dependent on dark‐induced [Ca^2+^]_cyt_ signals (Figs 8b,c, S10). Finally, we investigated the control of [Ca^2+^]_cyt_ in DD in the presence of sucrose, because sucrose sustains circadian rhythms in DD (Dalchau *et al.*, 2011). When sucrose was included in the media, we detected sustained circadian low‐amplitude oscillations of [Ca^2+^]_cyt_ in DD (period = 27.4 h, RAE = 0.59) (Figs 8d, S10), in which there are no light‐to‐dark transitions. These oscillations of basal [Ca^2+^]_cyt_ in DD occurred in the absence of dark‐induced [Ca^2+^]_cyt_ transients (Figs 8d, S10), demonstrating that a light‐to‐dark transition is not the cause of basal oscillations of [Ca^2+^]_cyt_. It also shows that sucrose can have different effects on [Ca^2+^]_cyt_ in the light and in the dark. In LL conditions, sucrose abolishes circadian oscillations of [Ca^2+^]_cyt_ (Johnson *et al.*,^1995^), whereas in DD conditions rhythms are sustained. All these results suggest that circadian signalling is involved in the regulation of both daily and circadian oscillations of [Ca^2+^]_cyt_ and the [Ca^2+^]_cyt_ changes that occur upon a light‐to‐dark transition.

## Discussion

In several previous studies, [Ca^2+^]_cyt_ changes upon transition to darkness have not been detected using either the luminescent reporter protein aequorin (Nomura *et al.*, 2012; Sello *et al.*, 2016; Frank *et al.*, 2019) or the ratiometric reporter protein Yellow Cameleon YC 3.6 (Loro *et al.*, 2016), despite the fact that they were previously reported by Sai & Johnson (2002). Therefore, it has been a matter of conjecture whether they occur or whether they cannot be resolved. Here we demonstrate that light‐to‐dark transitions generate a consistent and reproducible transient in [Ca^2+^]_cyt_. We used an ICCD225 photon‐counting camera that optimized the detection of aequorin luminescence from the leaves specifically and also because, compared with luminometer experiments, using a camera allows the signal‐to‐noise ratio to be increased by focusing the light from a number of plants onto a small area on the array and using time series integration. Thus, even though luminometers are more sensitive photon‐counting devices, using a camera permitted us to detect, at the end of the photoperiod, a prolonged increase in [Ca^2+^]_cyt_. To ensure that the signal we were measuring did not originate from the stroma, we compared it with measurements of changes in [Ca^2+^]_stroma_ after transition to darkness (Sai & Johnson, 2002; Nomura *et al.*, 2012; Sello *et al.*, 2016). The [Ca^2+^]_cyt_ transient consists of a peak at *c.* 10 min after the onset of darkness and a sustained increase that peaks at 30 min after the onset of darkness which decays over the next 2 h, whereas the [Ca^2+^]_stroma_ consists of two peaks at *c.* 8 and 25 min, lasting 40 min, and a prolonged increase that lasts *c.* 2 h after the onset of darkness (Fig. 1c). The [Ca^2+^]_stroma_ signature upon darkness obtained by the photon‐counting camera was slightly different from the one reported in previous studies (Sai & Johnson 2002; Nomura *et al.*, 2012; Sello *et al.*, 2016, 2018) and also here using a luminometer (Fig. S3). Using an intensified photon‐counting camera as the detector allowed higher resolution in the [Ca^2+^]_cyt_ and [Ca^2+^]_stroma_ response to this stimulus than with a luminometer, describing more complex [Ca^2+^] dynamics in both compartments. These results highlight the importance of the detection methods when aequorin is the reporter, and reveal that even though the dark‐induced [Ca^2+^]_cyt_ signal was detected in the luminometer, we were close to the detection limit (Fig. S4).

Sunset and shading are two environmental stimuli that are represented by the transition to darkness. We have observed that, similar to the dark‐induced [Ca^2+^]_stroma_ changes (Sai & Johnson, 2002), [Ca^2+^]_cyt_ signatures upon darkness are affected by the duration of the photoperiod (Fig. 4), suggesting that these [Ca^2+^]_cyt_ changes could be a mechanism by which plants can distinguish between long and short days and thereby induce photoperiodic responses. The gating of the signal by the time of day (Fig. 5) and circadian oscillator (Fig. 6) might suggest that the [Ca^2+^]_cyt_ changes after transition to darkness also encode information about the time of day. Additionally, we found that the [Ca^2+^]_cyt_ changes after transition to darkness are specific for photosynthetic organs, which are normally in the light (Fig. 2) and that [Ca^2+^]_cyt_ changes upon darkness involve at least the *PHYA* photoreceptors (Fig. 3c). The finding about the involvement of *PHYA* in the dark‐induced [Ca^2+^]_cyt_ signal is supported by our previous studies of circadian regulation of Ca^2+^ (Dalchau *et al.*, 2010) and by the maximum expression of the *PHYA* gene close to the end of the light interval, which is characteristic of light signals that regulate important physiological responses (e.g. end‐of‐the‐day far‐red response) (Toth *et al.*, 2001).

Additionally, the comparison of the [Ca^2+^]_cyt_ traces recorded when Ga^3+^ or La^3+^ were added before the transition into darkness (Fig. 3) suggests that the dark‐induced cytosolic [Ca^2+^]_cyt_ increase may be derived from internal stores rather than extracellular space. Furthermore, the diminution/abolishment of the [Ca^2+^]_cyt_ upon darkness in the presence of cPTIO and nicotinamide, which inhibit ADPRc activity (Fig. 3), might involve the vacuole and the ER in the generation/intensity of the [Ca^2+^]_cyt_ signal, similar to [Ca^2+^]_cyt_ circadian oscillations (Dodd *et al.*, 2007). The source of the dark‐induced [Ca^2+^]_stroma_ spike has been a matter of conjecture. It has been suggested that it is not dependent on photosynthetic electron transport because DCMU had little or no effect on the magnitude of the dark‐stimulated Ca^2+^ flux (Sai & Johnson, 2002). Additionally, the knockout mutation of BICAT2, which mediates Ca^2+^ uptake across the chloroplast envelope, strongly dampens the dark‐induced [Ca^2+^]_stroma_ signal, suggesting a dark‐triggered influx of Ca^2+^ from the cytosol, mediated by this transporter (Frank *et al.*, 2019). In our experiments, inhibition of photosynthetic electron transport by DCMU (Fig. 3) had a negative effect on the dark‐induced [Ca^2+^]_cyt_ signal, suggesting that photosynthesis might be necessary for the plants to respond. We hypothesize that the mechanism by which photosynthesis dampens dark‐induced [Ca^2+^]_cyt_ signal could be associated with the effect of photosynthetic sugars on the abundance of oscillator components (Haydon *et al.*, 2013; Haydon *et al.*, 2017), which also regulate the dark‐induced [Ca^2+^]_cyt_ increase (Fig. 6). The decrease in the dark‐induced [Ca^2+^]_cyt_ signal by DCMU and the lack of effect on the [Ca^2+^]_stroma_, indicates that upon darkness the regulatory mechanisms of cytosolic and stromal [Ca^2+^] signatures might be distinct, as previously suggested by Sai & Johnson (2002) and Sello *et al.* (2018). This conclusion is also supported by the fact that the [Ca^2+^]_cyt_ signal is larger when plants were grown in LD vs SD conditions and, interestingly, the dark‐induced stromal [Ca^2+^] increase has a larger peak in SD than in LD conditions (Fig. 4).

A striking result of our study is that, upon darkness, not only [Ca^2+^]_cyt_ but also [Ca^2+^]_stroma_ signatures are under the control of the circadian clock (Fig. 7) because it was previously concluded that daily dark‐stimulated [Ca^2+^]_stroma_ spikes are not gated by the circadian clock (Sai & Johnson, 2002). The failure to detect gating of [Ca^2+^]_stroma_ signatures in the previous study might be because the dark‐induced signal was measured only after 5 d in LL conditions when the circadian system might have become damped. It has been suggested that dampening might be a result of the clock damping in all cells or cell desynchronization in terms of phase or period, or desynchronization as a result of stochasticity in clock activity (Komin *et al.*, 2011; Guerriero *et al.*, 2012).

Circadian [Ca^2+^]_cyt_ oscillations in Arabidopsis are robust in LL but absent in DD when sucrose is not included in the media (Johnson *et al.*, 1995; Love *et al.*, 2004); therefore, they are usually measured by performing with breaks of darkness every 2 h in otherwise LL conditions. Our discovery of the dependence on the time of the day of the dark‐induced [Ca^2+^]_cyt_ changes resulting from circadian‐gating, forced us to consider whether circadian and daily oscillations of basal [Ca^2+^]_cyt_ might arise as a consequence of repeated dark‐induced increases in [Ca^2+^]_cyt_, which might not occur in the absence of the measuring protocol. We conclude that there are two modes of circadian regulation of [Ca^2+^]_cyt_, the circadian‐gating of dark‐induced increases in [Ca^2+^]_cyt_ and 24 h oscillations in basal [Ca^2+^]_cyt_ that are not a consequence of transfer to darkness, commonly called circadian oscillations of [Ca^2+^]_cyt_ (Johnson *et al.*, 1995; Love *et al.*, 2004; Xu *et al.*, 2007). We reached this conclusion by calculating the total photon counts including and excluding the dark‐induced [Ca^2+^]_cyt_ signal in LL conditions and by measuring [Ca^2+^]_cyt_ in DD conditions in the presence and absence of sucrose (Fig. 8). The detection of circadian oscillations of [Ca^2+^]_cyt_ in DD conditions in the presence of sucrose demonstrates unequivocally that basal [Ca^2+^]_cyt_ can free run with a circadian period.

Since the initial discovery of basal circadian oscillations of [Ca^2+^]_cyt_ by Johnson *et al*. (1995), their purpose has been a mystery, because Ca^2+^ signalling is usually considered to work on timescales much shorter than circadian timescales. Our finding of similarities between circadian and dark‐induced increases in [Ca^2+^]_cyt_ suggests that they might have a common basis and function. Basal circadian oscillations of [Ca^2+^]_cyt_ and circadian‐gated dark‐induced [Ca^2+^]_cyt_ signals have their greatest magnitude near dusk, suggesting that their purpose could be associated with dusk sensing and/or day‐length sensing. This dusk‐associated timing is consistent with the sensing of circadian [Ca^2+^]_cyt_ signals by calmodulin‐like 24 Ca^2+^ sensors that genetically interact with TOC1 to form part of the circadian oscillator, because TOC1 is expressed maximally near dusk (Marti *et al.*, 2018). One possibility is that the oscillation of basal [Ca^2+^]_cyt_ is indicative of the relaxing of the ‘gate’ by which the circadian oscillator times dark‐induced increases in [Ca^2+^]_cyt_ at the end of the photoperiod. The existence of the dark‐induced [Ca^2+^]_cyt_ signal reported here and the methodology described to characterize it, together with the novel aequorin reporters for chloroplast subcompartments (Sello *et al.*, 2018) and the discovery of the two chloroplast‐targeted Ca^2+^ transporters in *Arabidopsis thaliana*, BIVALENT CATION TRANSPORTER 1 (BICAT1) and BICAT2 (Frank *et al.*, 2019), which determine the amplitude of the prolonged and sustained dark‐induced [Ca^2+^]_stroma_, will pave the way to understanding the function and unravel the mechanisms responsible for [Ca^2+^] fluxes during light‐to‐dark transitions. Here, our data also demonstrate that the circadian oscillator in the nucleus, which is of eukaryotic origin, can regulate the timing of stimulus‐induced increases of [Ca^2+^]_stroma_ in the chloroplast, which is of bacterial origin, suggesting that a mechanism has evolved that allows temporal information to be communicated between these two organelles, resulting in time of day‐dependent Ca^2+^ signals and adding a new subcellular spatial dimension to the circadian network of plants.

## Author contributions

MCMR and HJJ carried out the experiments and data analyses. MCMR and AARW conceived the research, designed the experiments and wrote the manuscript.

## Supporting information


**Fig. S1** Dark‐induced [Ca^2+^]_cyt_ changes upon darkness using a photon‐counting camera.
**Fig.** **S2** Dark‐induced [Ca^2+^]_cyt_ transient does not occur 6 h after light off or in nontransgenic plants in 12 h : 12 h light : dark cycles.
**Fig.** **S3** The signature of the dark‐induced [Ca^2+^]_stroma_ changes upon darkness depends on the detection method.
**Fig.** **S4** Calibration of the dark‐induced [Ca^2+^]_cyt_ changes upon darkness using a luminometer.
**Fig.** **S5** The dark‐induced [Ca^2+^]_cyt_ transient occurs in green tissues.
**Fig.** **S6** The dark‐induced [Ca^2+^]_cyt_ transient depends on the time of the day.
**Fig.** **S7** Dark‐induced increases in luminescence were not detected from plants not carrying the aequorin transgene.
**Fig.** **S8** The dark‐induced [Ca^2+^]_cyt_ transient is gated by the circadian clock.
**Fig.** **S9** The dark‐induced [Ca^2+^]_stroma_ transient is gated by the circadian clock.
**Fig.** **S10** Dark‐induced transients in [Ca^2+^]_cyt_ are superimposed on daily and circadian [Ca^2+^]_cyt_ oscillations.
Please note: Wiley Blackwell are not responsible for the content or functionality of any Supporting Information supplied by the authors. Any queries (other than missing material) should be directed to the *New Phytologist* Central Office.
