# Supplementary material for: Circadian gating of dark‐induced increases in chloroplast‐ and cytosolic‐free calcium in Arabidopsis
Source: New Phytol. 2019 Nov 22;225(5):1993–2005. doi: 10.1111/nph.16280 (PMC7028143; doi:10.1111/nph.16280)

New Phytologist

***New Phytologist* Supporting Information**

Article title: **Circadian gating of dark-induced increases in chloroplast- and cytosolic-free calcium in Arabidopsis**

Authors: María Carmen Martí Ruiz, Hyun Ju Jung and Alex A. R. Webb

Article acceptance date: 11 October 2019

The following Supporting Information is available for this article:

Fig. S1 Dark-induced $[Ca^{2+}]_{cyt}$ changes upon darkness using a photon counting camera

Fig. S2 Dark-induced $[Ca^{2+}]_{cyt}$ transient does not occur 6 after light off or in non-transgenic plants in 12h-12h light-dark cycles

Fig. S3 The signature of the dark-induced $[Ca^{2+}]_{stroma}$ changes upon darkness depends on the detection method

Fig. S4 Calibration of the dark-induced $[Ca^{2+}]_{cyt}$ changes upon darkness using a luminometer

Fig. S5 The dark-induced $[Ca^{2+}]_{cyt}$ transient occurs in green tissues

Fig. S6 The dark-induced $[Ca^{2+}]_{cyt}$ transient depends on the time of the day

Fig. S7 Dark-induced increases of luminescence were not detected from plants not carrying the Aequorin transgene

Fig. S8 The dark-induced $[Ca^{2+}]_{cyt}$ transient is gated by the circadian clock

Fig. S9 The dark-induced $[Ca^{2+}]_{stroma}$ transient is gated by the circadian clock

Fig. S10 Dark-induced transients in $[Ca^{2+}]_{cyt}$ are superimposed on daily and circadian $[Ca^{2+}]_{cyt}$ oscillations

Fig. S1 Dark-induced $[Ca^{2+}]_{cyt}$ changes upon darkness using a photon counting camera. Luminescence from reconstituted aequorin in Col-0 Arabidopsis seedlings expressing aequorin targeted to the cytosol. Data were recorded immediately after plants were transferred to darkness at the end of the photoperiod (12 h after the lights went on) as shown in the bar above figure. Each experiment represents the luminesce from 24 clusters of seedlings, each cluster contained 7 - 10 seedlings.

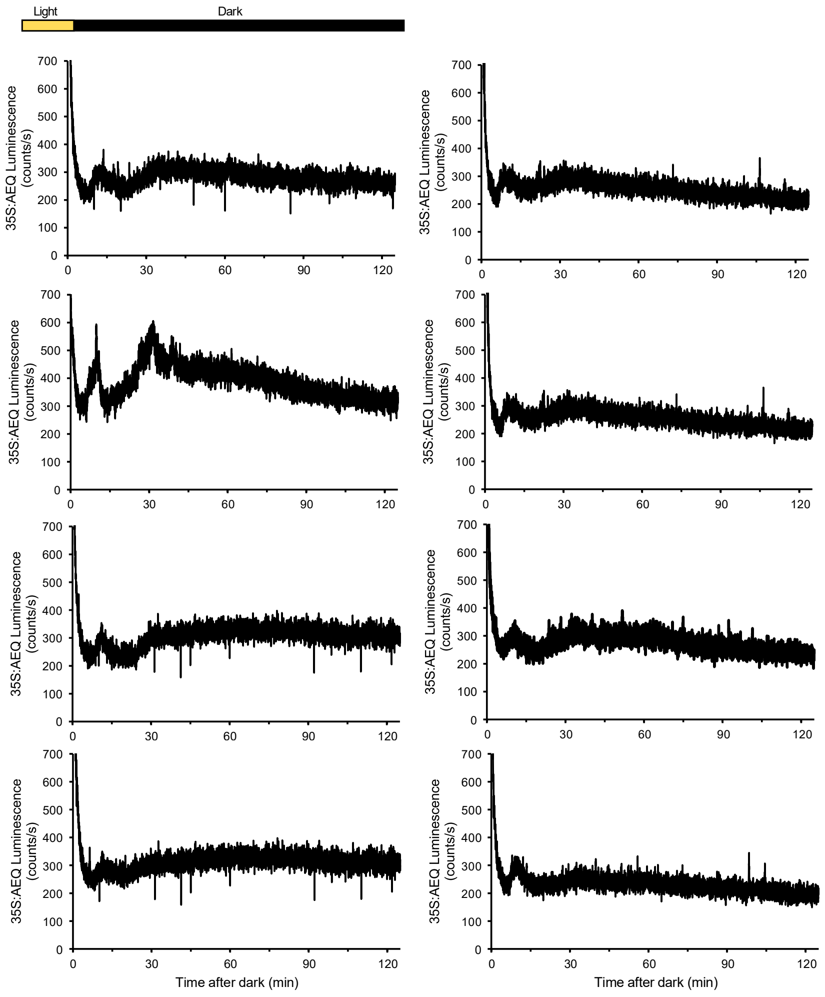

Fig. S2 Dark-induced $[Ca^{2+}]_{cyt}$ transient does not occur 6 after light off or in non-transgenic plants in 12h-12h light-dark cycles. Luminescence from reconstituted aequorin in Arabidopsis seedlings expressing aequorin targeted to the cytosol (black) (a) and from non-transgenic seedlings (blue) (b). The ecotype used was Col-0. Data were recorded immediately after plants were transferred to darkness at the end of the photoperiod (12 h after the lights went on) (b) or after being 6 h in the dark (a) as shown in the bars above the figures. Data represent the luminescence from one experiment consisting of 24 clusters of seedlings, each cluster contained 7 - 10 seedlings. These experiments are a repeat of the one showed in Fig. 1.

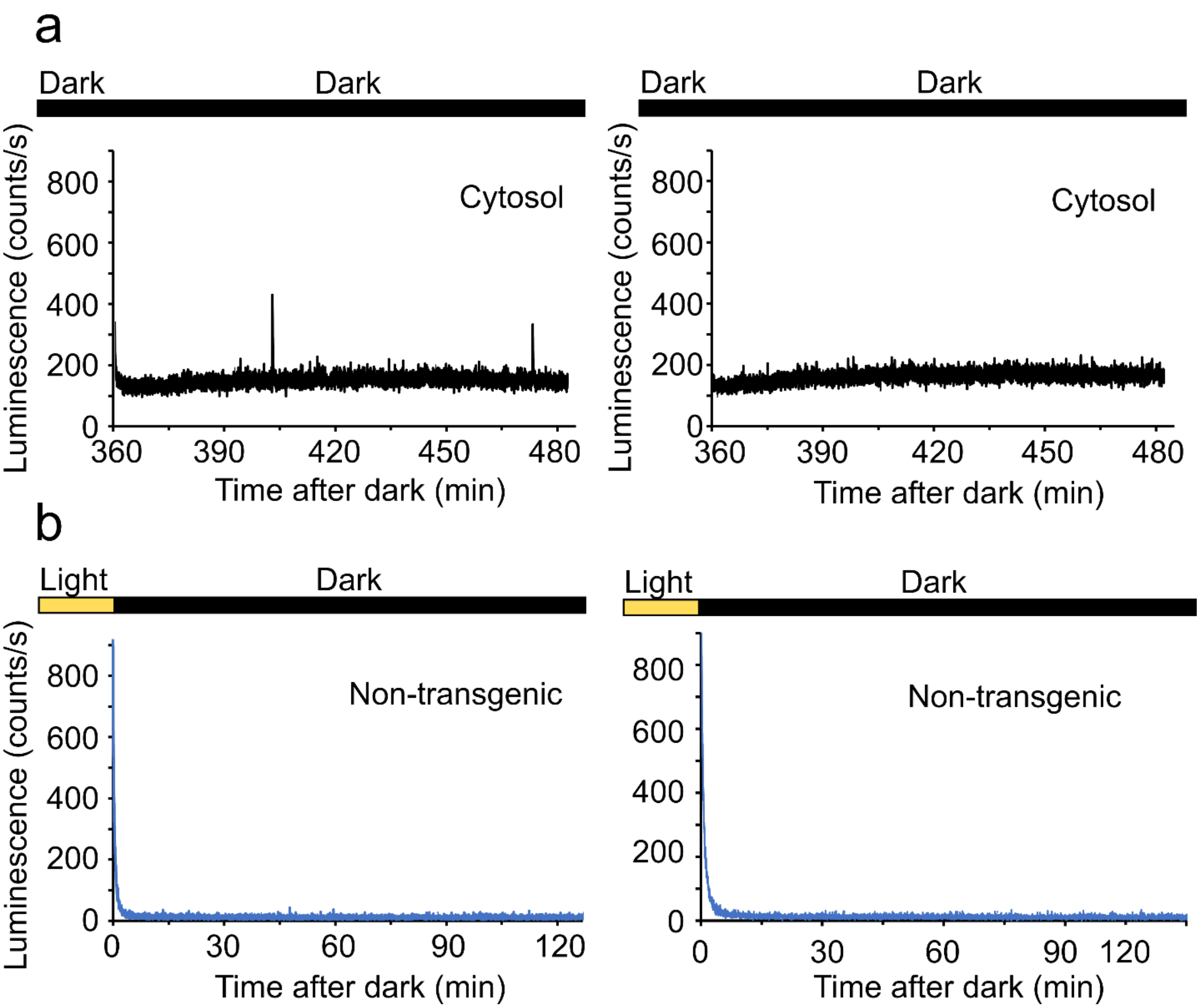

Fig. S3 The signature of the dark-induced $[Ca^{2+}]_{stroma}$ changes upon darkness depends on the detection method. Luminescence or $[Ca^{2+}]$ from reconstituted aequorin in Ws-2 Arabidopsis seedlings expressing aequorin targeted to the stroma. Data were recorded immediately after plants were transferred to darkness at the end of the photoperiod (12 h after the lights went on) as shown in the bars above the figures. Camera data represent the luminescence from 24 clusters of seedlings, each cluster contained 7 - 10 seedlings. Luminometer data represent the luminescence from three plants. Experiments were repeated at least twice and three are represented in the figure.

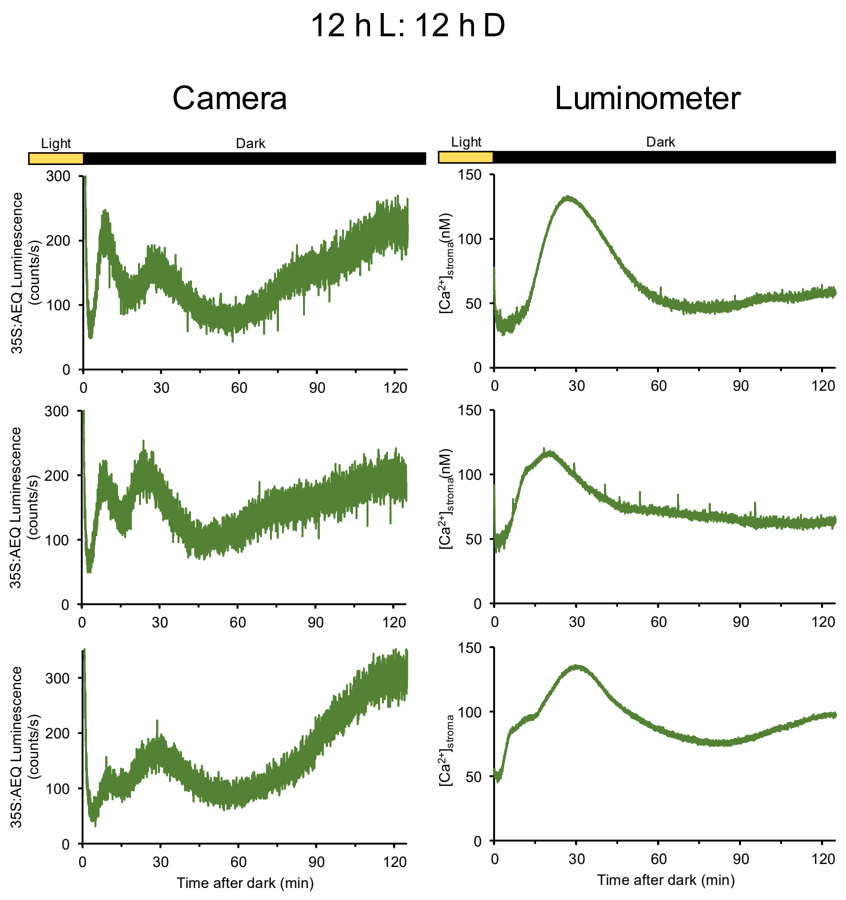

Fig. S4 Calibration of the dark-induced $[Ca^{2+}]_{cyt}$ changes upon darkness using a luminometer. $[Ca^{2+}]_{cyt}$ from reconstituted aequorin in Col-0 Arabidopsis seedlings expressing aequorin targeted to the cytosol. Data were recorded immediately after plants were transferred to darkness at the end of the photoperiod (12 h after the lights went on) as shown in the bars above the figures. Each graph represents the luminescence from three plants. Experiments were repeated 14 times with each trace presented in the figure.

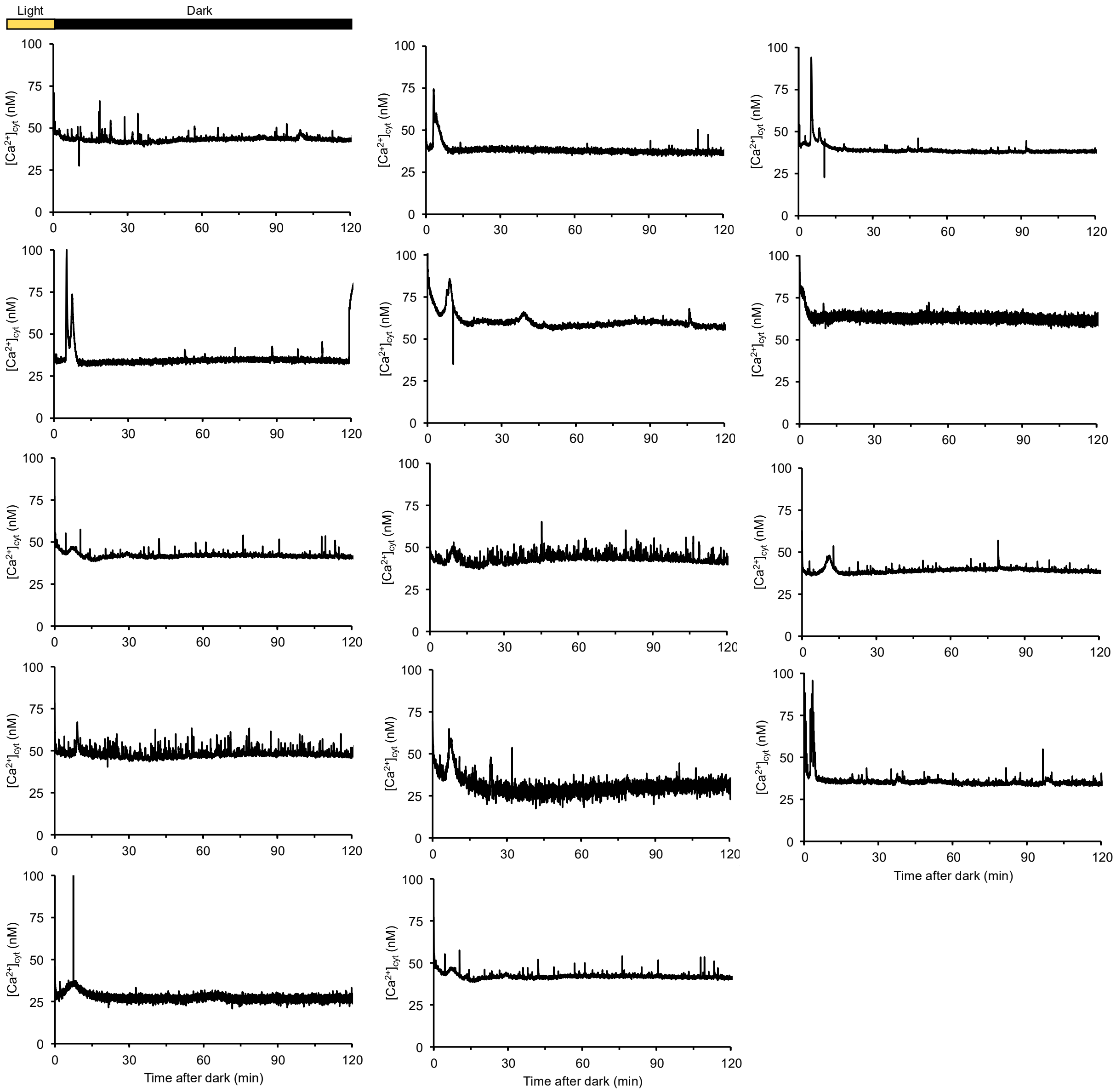
Light
Dark
[Ca2+]cyt (nM)
Time after dark (min)

Fig. S5 The dark-induced $[Ca^{2+}]_{cyt}$ transient occurs in green tissues. Three Col-0 Arabidopsis transgenic seedlings expressing aequorin targeted to the cytosol were grown in white light-dark cycles. On the night of the 11th day of growth, the root and leaves were separated and incubated with coelenterazine. Aequorin luminescence was recorded in a luminometer from reconstituted aequorin when tissues were 12 days old. Traces represent the data obtained from one experiment. This is a repeat of figure 2 experiment.

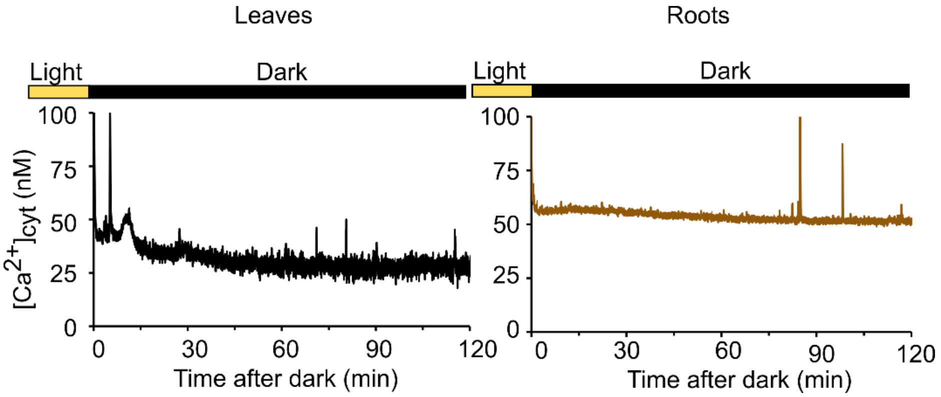

Fig. S6 The dark-induced $[Ca^{2+}]_{cyt}$ transient depends on the time of the day. Arabidopsis transgenic seedlings expressing aequorin targeted to the stroma (Ws-2) were grown in white light-dark cycles (12h:12h) (100 µmol m^{-2} s^{-1}). On the night of the 11th day of growth, seedlings were incubated with coelenterazine and aequorin luminescence was recorded from reconstituted aequorin since they were 12 days old. (A) shows data of changes in $[Ca^{2+}]_{stroma}$, every 2 h during 1 LD cycle. The data represent one experiment consisting of 80 clusters of seedlings and each cluster contained 7 - 10 seedlings. Experiments were repeated at least twice. This experiment is a repeat of the one in Figure 5b.

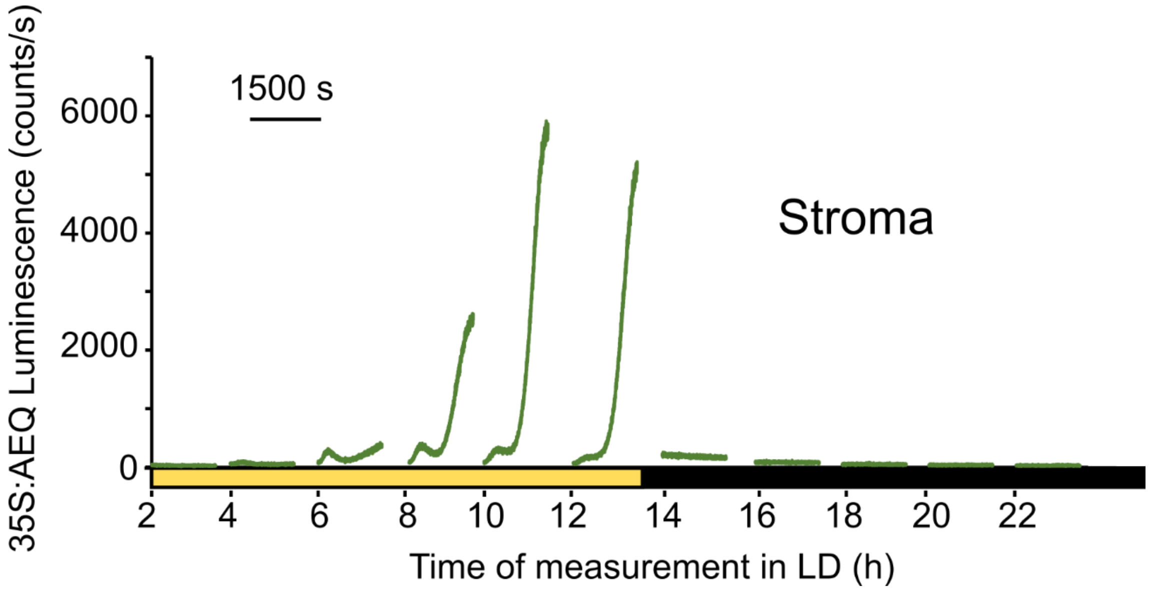

Fig. S7 Dark-induced increases of luminescence were not detected from plants not carrying the Aequorin transgene. Non-transgenic Col-0 Arabidopsis seedlings were grown in white light-dark cycles (12h:12h) (100 µmol m^{-2} s^{-1}). On the night of the 11th day of growth, seedlings were incubated with coelenterazine and luminescence was recorded when they were 12 days old. Graphs shows data during 1 LD 12h:12h cycle taken every 2 h during 1500 s. There were 80 clusters of seedlings per plate and each cluster contained 7 - 10 seedlings.

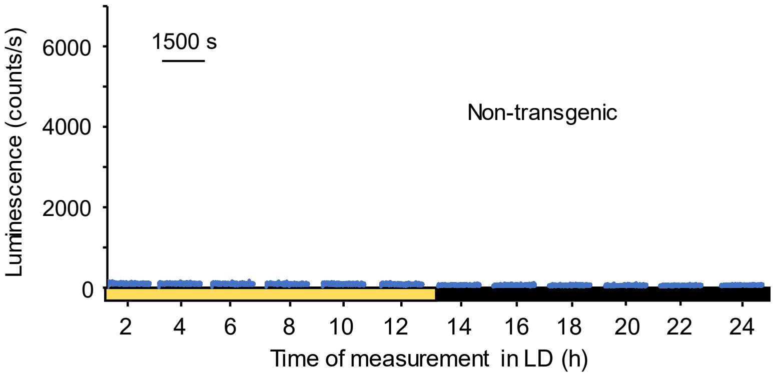

Fig. S8 The dark-induced $[Ca^{2+}]_{cyt}$ transient is gated by the circadian clock. Col-0 and *prr7*-11 *prr5*-10 *prr9*-11 triple mutant Arabidopsis transgenic seedlings expressing aequorin targeted to the cytosol were grown in white light-dark cycles (12h:12h) (100 µmol m^{-2} s^{-1}). On the night of the 11th day of growth, seedlings were incubated with coelenterazine and aequorin luminescence was recorded from reconstituted aequorin since they were 12 days old. Figures show data of changes in $[Ca^{2+}]_{cyt}$ every 2 h during LL cycles in Col-0 and *prr7*-11 *prr5*-10 *prr9*-11 triple mutant plants, respectively. The data represent one experiment consisting of 8 clusters of seedlings, each cluster contained 7 - 10 seedlings. Experiments were repeated at least twice. This is a repeat of the experiment in Figure 6.

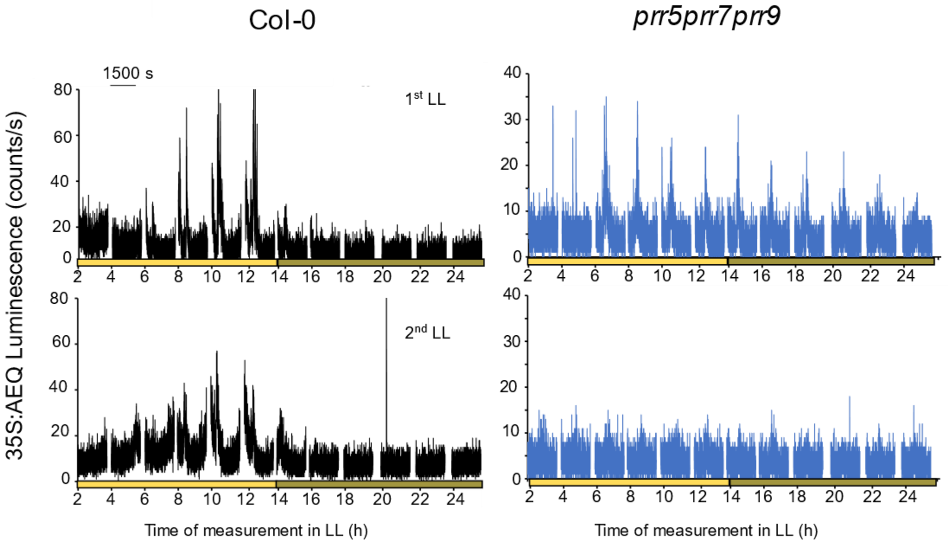

Fig. S9 The dark-induced $[Ca^{2+}]_{stroma}$ transient is gated by the circadian clock. Ws-2 Arabidopsis transgenic seedlings expressing aequorin targeted to the stroma were grown in white light-dark cycles (12h:12h) (100 µmol m^{-2} s^{-1}). On the night of the 11th day of growth, seedlings were incubated with coelenterazine and aequorin luminescence was recorded from reconstituted aequorin since they were 12 days old. The data show changes in $[Ca^{2+}]_{stroma}$ every 2 h during 2 LL cycles. The data represent one experiment consisting of 80 clusters of seedlings, each cluster contained 7 - 10 seedlings. This experiment is a repeat of the one in Figure 7.

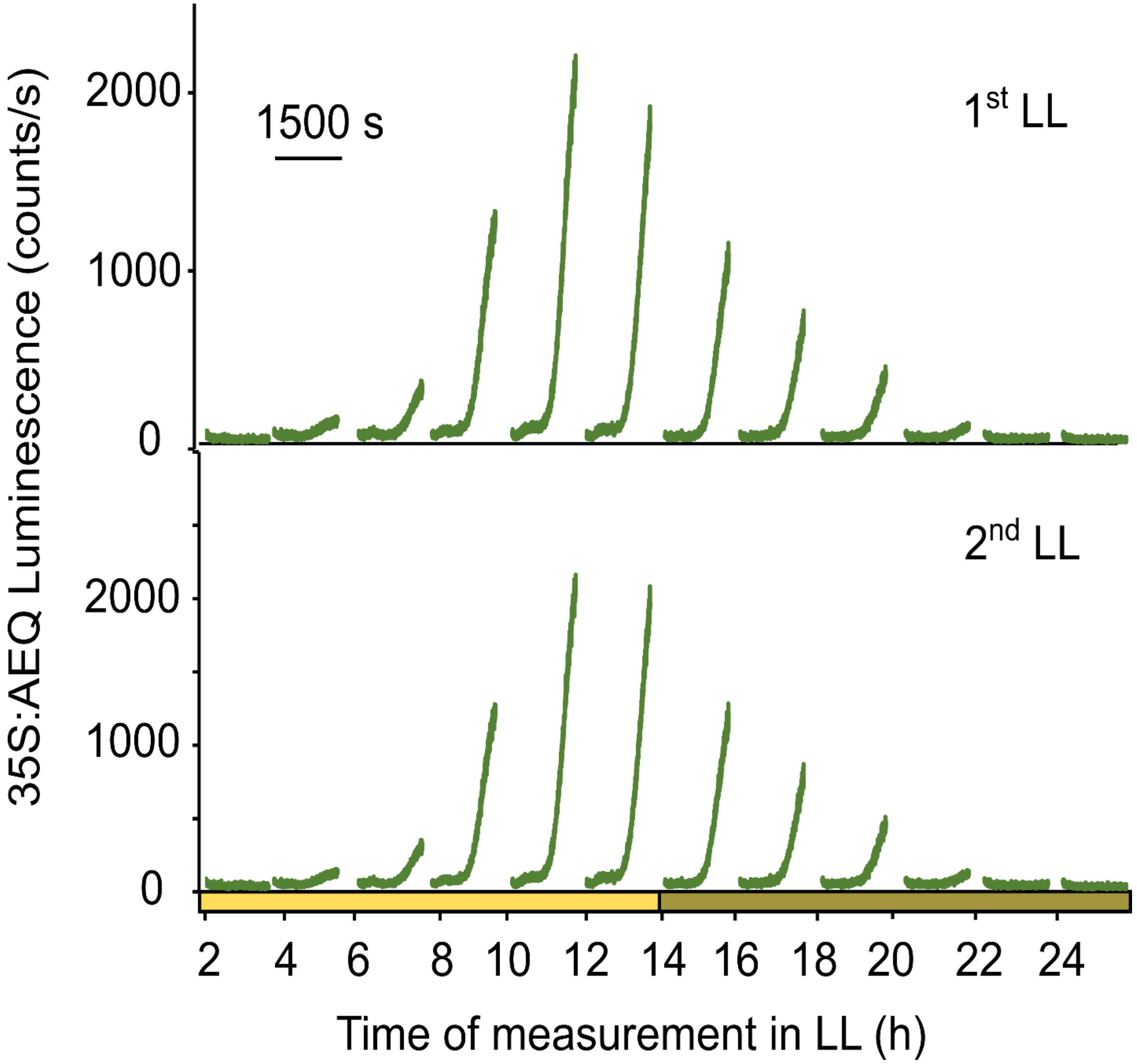

Fig. S10 Dark-induced transients in $[Ca^{2+}]_{cyt}$ are superimposed on daily and circadian $[Ca^{2+}]_{cyt}$ oscillations. (a) Daily and circadian $[Ca^{2+}]_{cyt}$ oscillations measured in LL using different time integration intervals. Col-0 Arabidopsis transgenic seedlings expressing aequorin targeted to the cytosol were grown in LD 12h:12h cycles and then transferred to LL. The data represent one experiment consisting of 80 clusters of seedlings and each cluster contained 7 - 10 seedlings. (b) Daily and circadian $[Ca^{2+}]_{cyt}$ oscillations measured in DD without (b) 3% (w/v) sucrose using different time integration intervals. (c) Changes in $[Ca^{2+}]_{cyt}$ recorded every 2 h and used in (B). (d) Daily and circadian $[Ca^{2+}]_{cyt}$ oscillations measured in DD with 3% (w/v) sucrose. Black are the raw time series data, showing no dark to light transitions. In red is the integrated photon counts obtained during the 1500 s of recording. (b-d) Col-0 Arabidopsis transgenic seedlings expressing aequorin targeted to the cytosol were grown in LD 12h:12h cycles and then transferred to DD. (b-d) the data represent one experiment consisting of 8 clusters of seedlings and each cluster contained 7 - 10 seedlings. These experiments are a repeat of the ones in Figure 8.

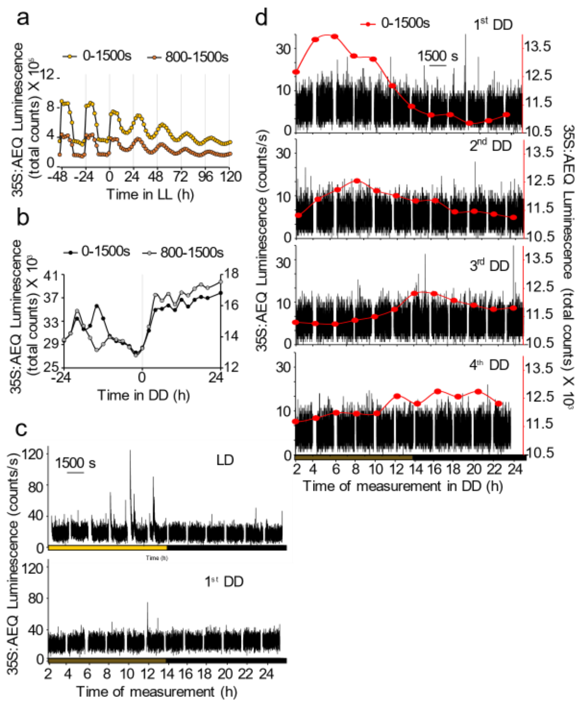

Supplement: Supplementary file 1 — Fig. S1 Dark‐induced [Ca2+]cyt changes upon darkness using a photon‐counting camera. Fig. S2 Dark‐induced [Ca2+]cyt transient does not occur 6 h after light off or in nontransgenic plants in 12 h : 12 h light : dark cycles. Fig. S3 The signature of the dark‐induced [Ca2+]stroma changes upon darkness depends on the detection method. Fig. S4 Calibration of the dark‐induced [Ca2+]cyt changes upon darkness using a luminometer. Fig. S5 The dark‐induced [Ca2+]cyt transient occurs in green tissues. Fig. S6 The dark‐induced [Ca2+]cyt transient depends on the time of the day. Fig. S7 Dark‐induced increases in luminescence were not detected from plants not carrying the aequorin transgene. Fig. S8 The dark‐induced [Ca2+]cyt transient is gated by the circadian clock. Fig. S9 The dark‐induced [Ca2+]stroma transient is gated by the circadian clock. Fig. S10 Dark‐induced transients in [Ca2+]cyt are superimposed on daily and circadian [Ca2+]cyt oscillations. Please note: Wiley Blackwell are not responsible for the content or functionality of any Supporting Information supplied by the authors. Any queries (other than missing material) should be directed to the New Phytologist Central Office. [file NPH-225-1993-s001.pdf]
